# Integrating Large Language Models with Deep Learning for Breast Cancer Treatment Decision Support

**DOI:** 10.3390/diagnostics16030394

**Published:** 2026-01-26

**Authors:** Heeseung Park, Serin Ok, Taewoo Kang, Meeyoung Park

**Affiliations:** 1 Department of Surgery, Biomedical Research Institute, Pusan National University Hospital, Busan 49241, Republic of Korea; paul0109@naver.com (H.P.); taewoo.d.kang@gmail.com (T.K.); 2 Department of Surgery, Pusan National University School of Medicine, Busan 49241, Republic of Korea; 3 Department of Computer Engineering, Kyungnam University, 7 Gyeongnamdaehak-ro, Masanhappo-gu, Changwon-si 51767, Republic of Korea; 2021111694@student.kyungnam.ac.kr

**Keywords:** breast cancer, large language model, clinical decision support system, pathology report, multi-label classification

## Abstract

**Background/Objectives**: Breast cancer is one of the most common malignancies, but its heterogeneous molecular subtypes make treatment decision-making complex and patient-specific. Both the pathology reports and the electronic medical record (EMR) play a critical role for an appropriate treatment decision. This study aimed to develop an integrated clinical decision support system (CDSS) that combines a large language model (LLM)-based pathology analysis with deep learning-based treatment prediction to support standardized and reliable decision-making. **Methods**: Real-world data (RWD) obtained from a cohort of 5015 patients diagnosed with breast cancer were analyzed. Meta-Llama-3-8B-Instruct automatically extracted the TNM stage and tumor size from the pathology reports, which were then integrated with EMR variables. A multi-label classification of 16 treatment combinations was performed using six models, including Decision Tree, Random Forest, GBM, XGBoost, DNN, and Transformer. Performance was evaluated using accuracy, macro/micro-averaged precision, recall, F1 score, and AUC. **Results**: Using combined LLM-extracted pathology and EMR features, GBM and XGBoost achieved the highest and most stable predictive performance across all feature subset configurations (macro-F1 ≈ 0.88–0.89; AUC = 0.867–0.868). Both models demonstrated strong discrimination ability and consistent recall and precision, highlighting their robustness for multi-label classification in real-world settings. Decision Tree and Random Forest showed moderate but reliable performance (macro-F1 = 0.84–0.86; AUC = 0.849–0.821), indicating their applicability despite lower predictive capability. By contrast, the DNN and Transformer models produced comparatively lower scores (macro-F1 = 0.74–0.82; AUC = 0.780–0.757), especially when using the full feature set, suggesting limited suitability for structured clinical data without strong contextual dependencies. These findings indicate that gradient-boosting ensemble approaches are better optimized for tabular medical data and generate more clinically reliable treatment recommendations. **Conclusions**: The proposed artificial intelligence-based CDSS improves accuracy and consistency in breast cancer treatment decision support by integrating automated pathology interpretation with deep learning, demonstrating its potential utility in real-world cancer care.

## 1. Introduction

Breast cancer is one of the most common solid tumors worldwide and represents a heterogeneous disease with diverse pathological characteristics [1]. Based on the expression of estrogen receptors (ER), progesterone receptors (PR), and human epidermal growth factor receptor 2 (HER2), breast cancer is classified into distinct molecular subtypes, each of which exhibits unique clinical behaviors and requires different therapeutic strategies [2,3,4]. HR-positive breast cancer expresses estrogen or progesterone receptors and responds well to endocrine therapy. By contrast, HER2-overexpressed breast cancer is characterized by rapid tumor growth resulting from excessive HER2 protein expression; however, this subtype demonstrates significant responsiveness to targeted therapies [5]. Because each subtype exhibits distinct biological behaviors and therapeutic responses, the treatment of breast cancer often involves a combination of surgery, chemotherapy, hormone therapy, targeted therapy, and radiation therapy [6,7]. These complex treatment decisions depend on the tumor stage, size, metastasis status, and molecular characteristics, requiring a precise interpretation of pathological information to ensure personalized treatment. Therefore, breast cancer treatment is highly sensitive and demands a precise medical approach that carefully reflects individual pathological and clinical characteristics.

In addition to the molecular subtype, the cancer stage plays a crucial role in determining the optimal treatment strategy for breast cancer. The stage is defined by pathological parameters such as tumor size (T), lymph node involvement (N), and the presence of distant metastasis (M), which are directly associated with prognosis and survival outcomes. However, in clinical practice, physicians must manually review a large volume of pathology reports to determine the cancer staging, which is both time-consuming and labor-intensive. Furthermore, differences in documentation styles and terminologies between hospitals often result in data inconsistency, which can reduce the reliability of stage determination. To address these challenges, there is an increasing need for an artificial intelligence (AI)-based system capable of automatically analyzing pathology reports and consistently extracting cancer staging information [8,9,10,11].

Recent advances in large language models (LLMs), a Transformer-based architecture trained on large-scale text data, have shown remarkable performance in natural language understanding and generation across various domains, including biomedical text mining and clinical data processing [12,13]. LLMs, such as GPT (Generative Pre-trained Transformer) [14], BERT (Bidirectional Encoder Representations from Transformers) [15], RoBERTa (Robustly Optimized BERT Approach) [16], and LLaMA (Large Language Model Meta AI) [17], have been successfully applied to unstructured medical texts to facilitate efficient information extraction and clinical decision support [18,19]. These models leverage self-attention mechanisms to capture long-range dependencies in complex narratives, which is particularly valuable when interpreting free-text pathology.

In this study, we utilized Meta-Llama-3-8B-Instruct, an instruction-tuned large language model developed by Meta AI [20]. Meta-Llama-3-8B-Instruct offers an optimal balance between computational efficiency and contextual understanding. Moreover, it has demonstrated strong zero-shot and few-shot reasoning capabilities, making it suitable for extracting clinical information from unstructured pathology reports [21]. Since Meta-Llama-3-8B-Instruct supports a maximum sequence length of up to approximately 80,000 tokens [22], it enables the model to effectively process long pathology texts without truncating important contextual information. This capability allows the model to achieve a more accurate and consistent extraction of cancer staging elements.

We aim to develop a clinical decision support system (CDSS) for breast cancer treatment by integrating LLMs with machine learning and deep learning techniques. This preliminary study presents an AI-driven framework that enhances the accuracy and reliability of breast cancer treatment planning using real-world clinical data.

## 2. Materials and Methods

### 2.1. Study Population

In this observational study, we analyzed real-world data (RWD) obtained from a cohort of 6175 patients diagnosed with breast cancer between 2011 and 2023 at Pusan National University Hospital (PNUH; 179 Gudeok-ro, Seo-gu, Busan, 49241, Republic of Korea). A diagnosis of breast cancer was defined as the presence of an International Classification of Diseases (ICD) code of either C50 (malignant neoplasm of the breast) or D05 (carcinoma in situ of the breast). The data were directly extracted from the EMRs of this tertiary referral hospital. As summarized in Table 1, twelve data domains were included, as follows: basic demographic patient information (*n* = 6175), outpatient visit records (*n* = 6171), inpatient visit records (*n* = 6008), diagnostic information (*n* = 6175), medication prescriptions (*n* = 6143), surgical prescriptions (*n* = 5685), treatment and procedures (*n* = 6169), laboratory tests (*n* = 6156), imaging and pathology reports (*n* = 6152), diagnostic tests (*n* = 6154), physical measurements (*n* = 5980), and vital signs (*n* = 5932).

This study was conducted in accordance with the Declaration of Helsinki, reported according to the Strengthening the Reporting of Observational Studies in Epidemiology statement, and approved by the Institutional Review Boards PNUH and PNUYH, which waived the consent for this study (IRB No. H-2401-032-135, 2024/02/02). Patient consent was waived due to the data for this study being de-identified and based on longitudinal observational health data.

### 2.2. Study Design and Cohort Construction

**Figure 1:**
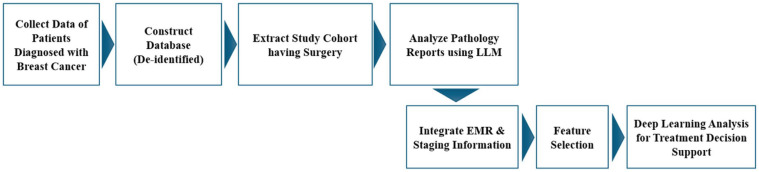
Overall data analysis workflow. The collected data were de-identified and securely stored in a protected database. Only patients who underwent surgery were included in the study cohort. Pathology reports were analyzed using a large language model to extract the staging information. The extracted stage data were then integrated with the electronic medical record data. Finally, deep learning analyses were performed to identify the treatment methods.

The overall study design is illustrated in Figure 1. To ensure patient privacy and data security, all identifiable information was removed, and a protected database was constructed. From this dataset, 5942 patients with available pathology reports were selected to establish the study cohort for the subsequent analysis. The pathology reports were then analyzed using an LLM to extract the staging information. The extracted staging data were integrated with the EMR data. Finally, deep learning analyses were conducted to identify the treatment methods (Figure 1).

**Figure 2:**
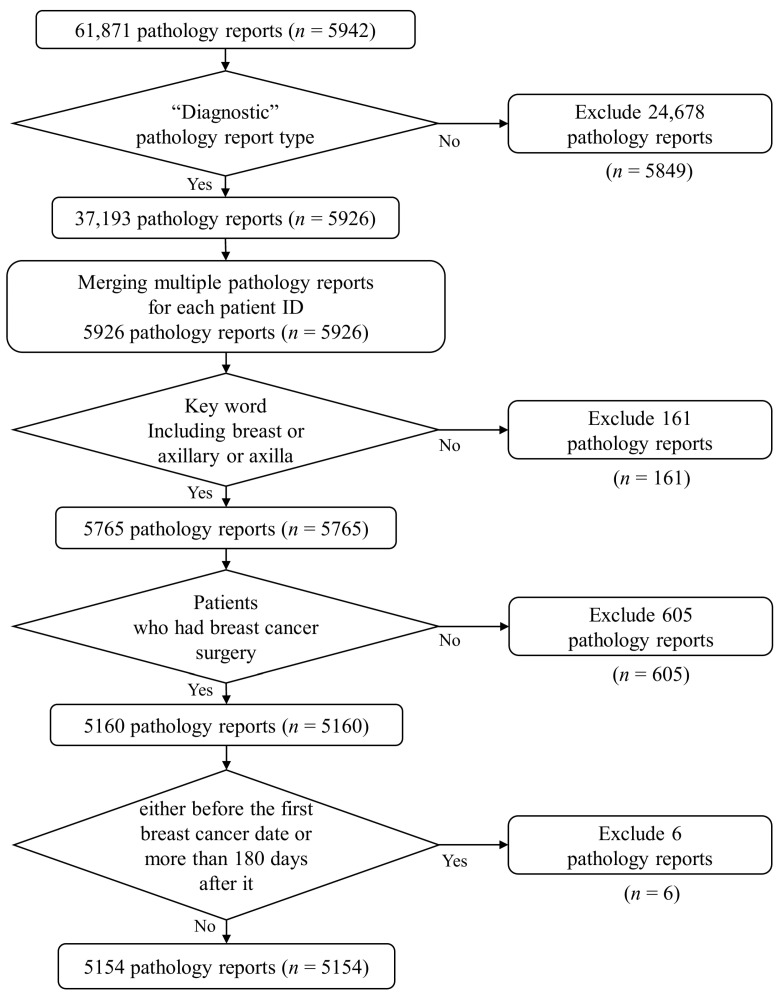
Flowchart of the cohort selection process. A total of 61,871 pathology reports were extracted and filtered through inclusion and exclusion criteria. Final cohort (*n* = 5154) was selected based on pathology report availability, breast cancer-related surgery, and report timing within 180 days of first surgery.

As shown in Figure 2, the pathology reports were extracted for the purpose of staging information extraction. From a total of 61,871 pathology reports obtained from 5942 patients, 37,193 diagnostic pathology reports were selected for further analysis, while 24,678 gross examination pathology reports without cancer staging information were excluded. For patients with multiple pathology reports, all reports were chronically merged into a single record. Next, only those pathology reports containing the keywords “Breast”, “Axillary”, or “Axillar” were retained (*n* = 5765), while reports related to other organs or unrelated to breast cancer were excluded. Subsequently, those patients who underwent surgeries unrelated to breast cancer were excluded. Finally, those pathology reports from surgeries performed before the first breast cancer-related surgery after the initial diagnosis and those created long after the first surgery were removed.

After applying these inclusion and exclusion criteria, a total of 5154 patients with pathology reports generated within 180 days of their first breast cancer-related surgery were included in the final study cohort. Figure 2 shows a detailed flowchart of the cohort selection process.

### 2.3. Pathology Report Analysis Using LLM

Our pathology reports associated with breast cancer-related surgeries were analyzed using an LLM to extract the tumor staging information. The LLM was prompted with structured templates designed to identify key pathological entities, including TNM stage, tumor size, ER, PR, and HER2. To extract the structured TNM staging information from the pathology reports, we employed Meta-Llama-3-8B-Instruct, an instruction-tuned transformer-based large language model. This model was selected because it provides an optimal balance between computational efficiency and contextual comprehension, which allows for accurate information extraction.

**Figure 3:**
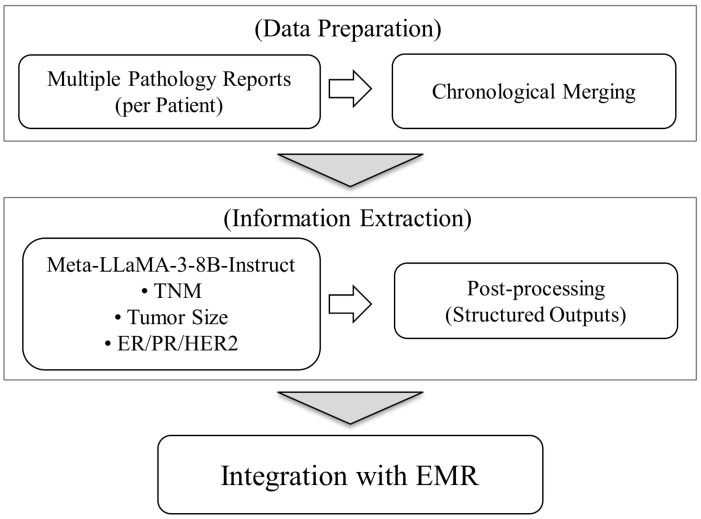
Workflow of the LLM-based pathology information extraction pipeline. Multiple pathology reports per patient are chronologically merged and processed by Meta-LLaMA-3-8B-Instruct using a predefined prompt to extract the TNM stage, tumor size, and hormone status. The structured outputs are post-processed and integrated with the EMR data for downstream modeling.

For each patient, multiple pathology reports were chronologically merged using date-based delimiters, and the report section closest to the surgery date was selected as the input. The selected text was processed by Meta-LLaMA-3-8B-Instruct with a predefined prompt to extract the TNM stage, tumor size, ER/PR, and HER2 status, as shown in Figure 3. The model outputs were post-processed to retain only the structured values and subsequently integrated with the existing EMR data at the patient level. Pathology reports often contain long and complex sentence structures, multiple diagnostic observations, and interdependent contextual cues. To address these challenges, we utilized the model’s 8192-token context window, which minimizes truncation and preserves complete contextual information. This configuration allows the model to process entire pathology narratives within a single inference window and ensures accurate and consistent extraction of the TNM staging elements across diverse report formats.

To improve accuracy and consistency, multiple prompt–response iterations were applied. The LLM output was validated by cross-checking with a subset of manually reviewed pathology reports, and the extracted staging information was further verified by clinical experts with expertise in breast cancer. Ambiguous or incomplete responses were manually curated and re-processed through refined prompts based on expert feedback. The final structured outputs were integrated with the corresponding EMR data to construct a comprehensive dataset reflecting both pathological and clinical characteristics. This integrated dataset subsequently served as input for machine learning and deep learning models developed to predict and support treatment decision-making (Figure 3).

The model performs information extraction without any prior examples and relies solely on explicit task instructions and output schema definitions. We designed prompts that included the following:

(1) A clear task definition (e.g., “start with the corresponding letter (e.g., ‘T’, ‘N’)”);
(2) Term interpretation rules (e.g., mapping expressions “TNM staging: pT1c pN2”);
(3) Uncertain value handling (e.g., impossible TNM value appears (e.g., “T10”, “N99”);
(4) A standardized output format based on the National Comprehensive Cancer Network (NCCN) guidelines [23].

### 2.4. Treatment Decision Support System for Breast Cancer

We developed a multi-label classification system to predict and support breast cancer treatment decisions by integrating pathology data with the EMR information. Unlike a single-label classification, which assigns each sample to one exclusive class, a multi-label classification allows for a single patient to belong to multiple treatment categories simultaneously. This approach more accurately represents the complexity of real-world breast cancer treatment, where treatment plans often involve combinations of therapies rather than a single modality.

The overall multi-label classification procedure consisted of four main stages, as follows:

(1) Multi-class labeling: each patient record was labeled according to the corresponding treatment method.
(2) Feature selection: the importance of each feature was evaluated to identify the most influential factors.
(3) Model training: machine learning and deep learning algorithms were applied to learn multi-label dependencies among treatment combinations.
(4) Model evaluation: the model performance was assessed using standard multi-label metrics—accuracy, macro- and micro-averaged precision, recall, and F1 score—to verify the reliability of predictions.

#### 2.4.1. Multi-Label Classification

To perform multi-label classification, four major treatment categories were defined as follows: chemotherapy, anti-hormone therapy, HER2-targeted therapy, and radiotherapy. For each patient, the presence or absence of each treatment record was encoded as a binary label (1 = received, 0 = not received). The four binary variables were then combined to generate composite label vectors representing individual treatment combinations. This labeling process defined 16 treatment classes, including no treatment, each representing a unique combination of the four therapy types, as summarized in Table 2.

#### 2.4.2. Feature Selection

Feature selection was performed to identify the predictive features for breast cancer treatment classification. A total of 9354 features were derived from the pathology reports and EMR data, including demographic (*n* = 4), diagnosis-related (*n* = 4944), drug-related (*n* = 4331), lab test (*n* = 67), and pathology-derived features (*n* = 8). Feature importance scores were computed using the MultiOutputClassifier module with a RandomForestClassifier as the base estimator.

Hyperparameters were configured, as summarized in Table 3, and a grid search approach was applied to optimize model performance. Feature importance values were obtained from the trained Random Forest model and ranked according to their relative contribution. Subsequently, based on these scores, cumulative feature subsets corresponding to the top 10% (*n* = 935), 20% (*n* = 1871), and 30% (*n* = 2806) of input variables were constructed, such that each larger subset included all features (*n* = 9354) from the smaller subsets, and were used for the subsequent comparative analysis.

#### 2.4.3. Deep Learning Analysis

For the multi-label classification, six representative models—Decision Tree [24], Random Forest [25], Gradient Boosting Machine (GBM) [26], XGBoost [27], Deep Neural Network (DNN) [28], and Transformer [29]—were employed to provide a comprehensive performance comparison across different learning paradigms. Tree-based models, such as Decision Tree, ensemble-based Random Forest, XGBoost, and GBM, were chosen for their robustness in handling heterogeneous tabular data, interpretability, and proven performance in previous medical data classification studies [30,31,32].

However, conventional ensemble methods often rely on global performance-based weighting schemes that ignore instance-level difficulty. To address this limitation, item response theory (IRT)-based ensembles model both classifier ability and sample difficulty, enabling instance-aware weighting that improves performance, particularly on hard-to-classify instances [8]. More recently, ensemble principles have been extended to large language models through LLM-Forest, an ensemble framework designed for LLM-based data imputation that adopts a Random Forest-like structure [33]. These studies demonstrate that incorporating instance difficulty, contextual relevance, and reliability into ensemble aggregation is critical for enhancing robustness across both traditional machine learning and LLM-based systems. Although IRT-based ensembles improve the accuracy of a weighted majority voting algorithm, they require additional response-matrix construction and parameter estimation, which substantially increase modeling complexity and implementation overhead. Since the primary objective of this study is robust and reproducible predictive performance, we adopt the Python (Version 3.10.16) RandomForestClassifier as a well-established ensemble method with strong empirical reliability and stable generalization behavior.

**Figure 4:**
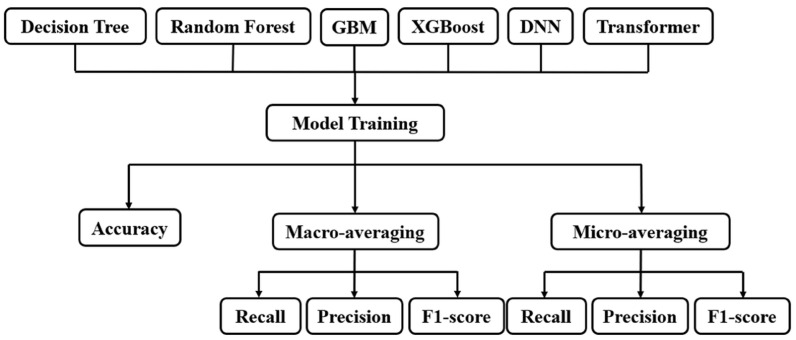
Deep Learning analysis workflow. Six classification models—Decision Tree, Random Forest, Gradient Boosting Machine, XGBoost, Deep Neural Network, and Transformer—were trained using the same integrated clinical–pathological dataset. Model performance was evaluated using accuracy and macro- and micro-averaged precision, recall, and F1 score to comprehensively assess the predictive performance across multi-class treatment labels.

A multilayer perceptron-based DNN was implemented to model complex and high-dimensional relationships between clinical and pathological variables, whereas the Transformer model was selected for its ability to capture long-range dependencies through self-attention mechanisms [34]. Each model was trained using the predefined hyperparameters summarized in Table 3, and the Hamming loss function was adopted as the main optimization objective to ensure consistency across all algorithms. Figure 4 illustrates the overall structure of the model training and evaluation process. After training six different models, performance metrics were computed separately for instance-based accuracy and for macro- and micro-averaged precision, recall, and F1 score to ensure balanced evaluation across all treatment labels.

#### 2.4.4. Model Training

The dataset was randomly divided into training (60%), validation (20%), and test (20%) subsets. Model training and evaluation were independently performed on four datasets: the full integrated dataset and three feature subsets corresponding to the top 10%, 20%, and 30% of feature importance scores. The purpose of this approach was to compare model performance across the feature subset to examine whether a smaller, more informative set of features could achieve comparable or superior predictive accuracy while reducing computational cost and model complexity. This analysis aimed to identify an optimal feature range that captures key determinants of breast cancer treatment decisions without relying on the entire dataset.

To ensure optimal performance and consistency across the models, hyperparameter optimization was conducted for all six algorithms: Decision Tree, Random Forest, GBM, XGBoost, DNN, and Transformer. Table 4 summarizes the key parameters for each model. For the tree-based models, hyperparameters such as max_depth, min_samples_split, and min_samples_leaf were tuned to balance model complexity and overfitting. The ensemble models (Random Forest, GBM, and XGBoost) were configured with numbers of estimators (300–700) and learning rates (0.1–0.01) to optimize generalization performance.

For the neural network models, both DNN and Transformer were trained for up to 3000 epochs with learning rates between 0.001 and 0.0001. The DNN architecture varied in hidden layer dimensions (64–256) and dropout rates (0.1–0.4) to prevent overfitting. The Transformer model was designed with different model dimensions (d_model = 64–128) and attention heads (nhead = 2–8) to evaluate the effect of self-attention configuration on classification performance. All models were trained using the Hamming loss as the primary objective function, and the configuration that achieved the lowest validation error was selected as the best-performing model.

#### 2.4.5. Model Evaluation

To evaluate the performance of the multi-label classification models, standard metrics, including accuracy, precision, recall, and F1 score, were applied. Because each patient could have multiple treatment labels simultaneously, label imbalance and overall predictive consistency were addressed using both macro-averaging and micro-averaging strategies for precision, recall, and F1 score [35]. For multi-label classification, accuracy was calculated at the instance level. This metric represents the proportion of correctly predicted label sets for each patient. However, precision, recall, and F1 score were evaluated using two averaging strategies: macro-average and micro-average. In the macro-average approach, each metric was calculated independently for every class, and the unweighted mean was then taken across all classes. This method evaluates how well the model performs for each class, including those with fewer samples. In the micro-average approach, true positives, false positives, and false negatives were summed across all classes before precision, recall, and F1 score were computed. This method evaluates the overall predictive performance of the model, with greater influence from classes that occur more frequently. In this study, due to the substantial class imbalance across the 15 treatment combinations, the macro-averaging approach was selected as the primary evaluation method. This strategy provides a more reliable assessment of model performance under class imbalance.

## 3. Results

The primary objective of this study was to develop an integrated CDSS that combines an LLM-based extraction of pathological staging information with a deep learning-based treatment decision model. The dataset used in this system was prepared in two stages. First, cancer staging information was automatically extracted from unstructured pathology reports using the LLM. Second, the extracted pathological data were integrated with patient-level EMRs to construct a comprehensive dataset. Based on this integrated dataset, six multi-label classification models—Decision Tree, Random Forest, GBM, XGBoost, DNN, and Transformer—were trained to predict breast cancer treatment strategies. The predictive targets were defined as 16 distinct classes, each representing unique combinations of four therapeutic methods, including chemotherapy, anti-hormone therapy, HER2-targeted therapy, and radiotherapy.

### 3.1. Cohort Characteristics

**Figure 5:**
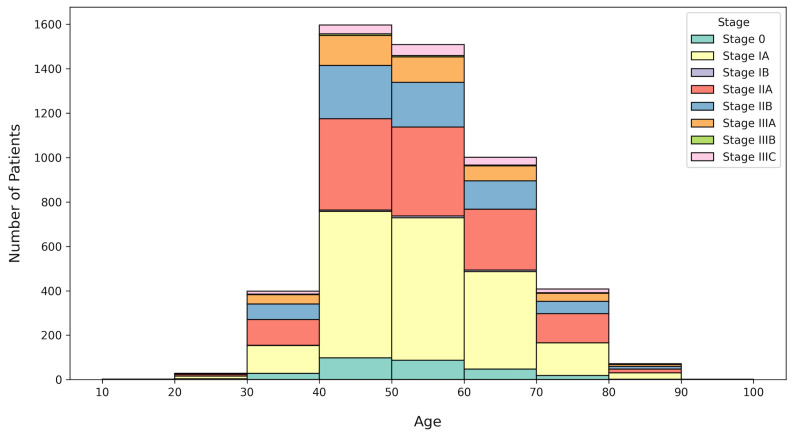
Distribution of breast cancer patients by age and clinical stage. The majority of patients were within the 40–60 age range, which shows the highest prevalence among middle-aged women. Early-stage breast cancer (Stage I–II) was most common, whereas advanced stages were relatively less frequent. Stage IV (metastatic cases) was excluded from visualization because metastatic records were not included in the analysis.

Breast cancer patients were stratified by age group and clinical stage according to the AJCC Cancer Staging Manual, 8th Edition [36], as summarized in Table 5 and Figure 5. The cohort included patients aged between 10 and 90 years, with the majority predominantly distributed in the 40–60 age range. Specifically, patients aged 40–50 years (*n* = 1597) and 50–60 years (*n* = 1508) accounted for the largest proportions of the study cohort. This age distribution reflects the well-known epidemiological trend that breast cancer prevalence peaks among middle-aged women.

Regarding cancer staging, early-stage breast cancer (Stage I and Stage II) was the most prevalent, whereas advanced stages (Stage IIIA–IIIC) were less frequent. Among the cohort, Stage IA (*n* = 2051) and Stage IIA (*n* = 1358) were the most common, and this trend reflects the early detection patterns generally observed in hospital-based datasets with routine screening programs. A smaller number of cases were identified in Stage 0 (*n* = 287) and Stage IIIC (*n* = 171), indicating that carcinoma in situ and late-stage breast cancer were relatively small in this dataset. Overall, the age and stage distribution demonstrate that the majority of patients were diagnosed at an early to intermediate stage. Stage IV (metastatic breast cancer) was excluded from the analysis process.

**Figure 6:**
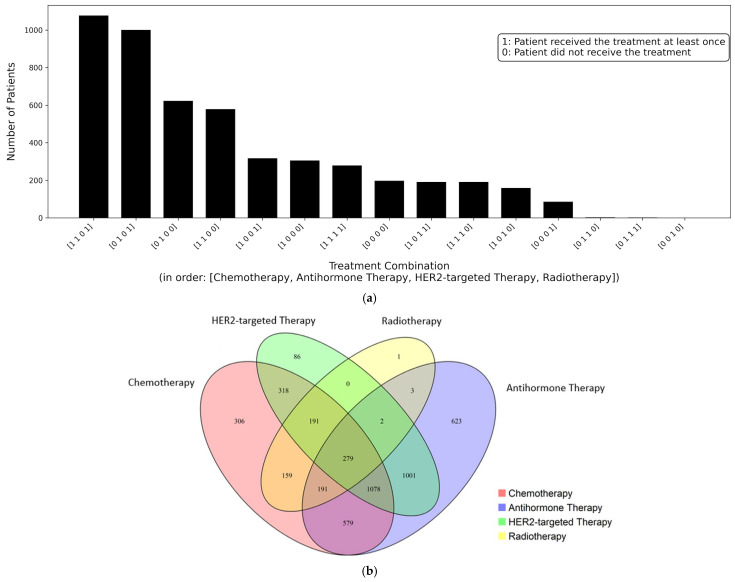
(**a**) Distribution of patients by treatment combination in multi-label classification. Each bar represents the number of patients corresponding to a specific treatment combination, expressed as a binary vector in the order of chemotherapy, anti-hormone therapy, HER2-targeted therapy, and radiotherapy (0: not received, 1: received). (**b**) Overlap of patients among four treatment types in breast cancer. The Venn diagram illustrates the number of patients receiving combinations of therapies. The overlapping areas represent patients who underwent multiple treatment types.

The distribution of treatment combinations is shown in Figure 6a. Among the 15 treatment combinations, chemotherapy and anti-hormone therapy were the most frequently used treatments, either alone or in combination with other therapies. This finding suggests that these two treatment types play a central role in the therapeutic strategies for breast cancer. Combinations receiving HER2-targeted therapy appeared less frequently, which is consistent with real-world clinical settings where patients with HER2-positive subtypes represent a smaller proportion of the overall breast cancer population. Radiotherapy was often applied in conjunction with other systemic treatments rather than as a standalone treatment.

The overlap among the four treatment types is shown in Figure 6b. The largest overlap was observed between chemotherapy and anti-hormone therapy. It suggests that these treatments are frequently administered together as part of a standard treatment plan. Overall, the distribution shows that many patients received combination-based clinical strategies that were personalized according to their pathological and molecular characteristics. Table 6 presents the number of patients in each of the 16 treatment combination classes within the study cohort. Among these, the class combining chemotherapy, anti-hormone therapy, and radiotherapy included 1078 patients, which represents one of the most common treatment combinations.

### 3.2. Evaluation of Pathology Information Extraction and Data Integration

Key clinical information, including the TNM stage, tumor size, ER/PR hormone receptor status, and HER2 status, was automatically extracted from the unstructured pathology reports using the proposed LLM-based pipeline. To evaluate the extraction performance, verification against the original pathology reports was required; however, full manual validation across all patients was not feasible due to substantial variability in the report formats. Instead, a subset of LLM-generated outputs was reviewed as part of a quality assurance process in collaboration with clinical experts by comparing the extracted information with the original pathology reports. This review confirmed that most extracted results were consistent with the source records, while a small number of cases with extraction errors were excluded from the subsequent analysis

The extraction performance was improved through iterative prompt refinement. The prompt was initially designed based on common reporting patterns in the pathology reports and subsequently revised through consultation with clinical experts to enhance clinical validity and consistency across the heterogeneous expression styles. Using the finalized prompt, the extracted outputs were post-processed to retain only the structured values and then integrated with the existing EMR data using patient identifiers. The integrated patient-level dataset was then used for downstream treatment prediction model training.

### 3.3. Feature Importance Analysis

A feature importance analysis revealed that hormone receptor-related markers (ER and PR) and HER2 status were the most influential variables in the multi-label classification model. These factors are clinically recognized as key determinants in establishing appropriate treatment strategies for breast cancer. In addition to these molecular biomarkers, several pathological variables extracted from the pathology reports, such as tumor size and lymph node involvement, were also found to be highly relevant to treatment prediction.

Demographic and physical characteristics—specifically age, height, and weight—were also identified as clinically meaningful predictors. These features are known to influence treatment decisions, as patient age and body composition often affect chemotherapy tolerance, hormonal therapy eligibility, and overall treatment planning.

**Figure 7:**
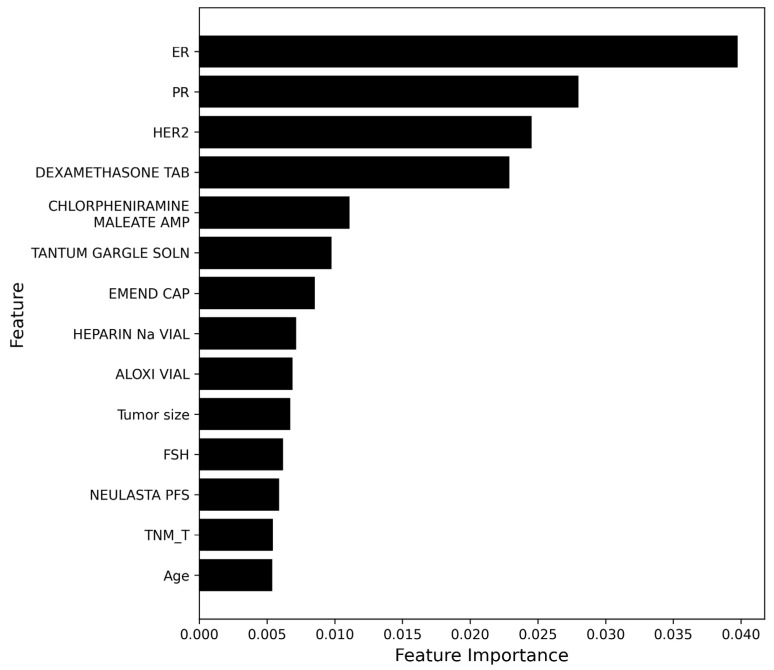
Top 15 features ranked by feature importance. The top 15 features based on feature importance scores derived from the Random Forest model. The importance values indicate the relative contribution of each feature to the breast cancer treatment classification task.

Figure 7 shows the top 15 features ranked by importance, as determined by the Random Forest model. These features exhibited higher contribution scores compared with other input features, and were identified as key predictors in the breast cancer treatment classification. The most highly ranked features primarily included pathology-derived variables related to tumor characteristics and staging information, as well as drug-related clinical features. The distribution of importance scores indicates that a subset of features accounted for a relatively larger contribution to model predictions.

Overall, the results demonstrate that the integration of pathological, physiological, and laboratory features provides a more clinically relevant system for breast cancer treatment decision support.

### 3.4. Comparative Performance Analysis of Multi-Label Classification Models

The dataset was randomly divided into training (60%), validation (20%), and test (20%) subsets for treatment classification. Table 7 shows the distribution of treatment combination classes across the training, validation, and test sets after dataset splitting. The samples were not uniformly distributed among the 16 treatment classes. A limited number of classes accounted for a substantial proportion of the dataset, whereas several classes contained relatively few samples. This distribution reflects the characteristics of real-world treatment data and indicates that class imbalance should be considered when interpreting classification results.

**Figure 8:**
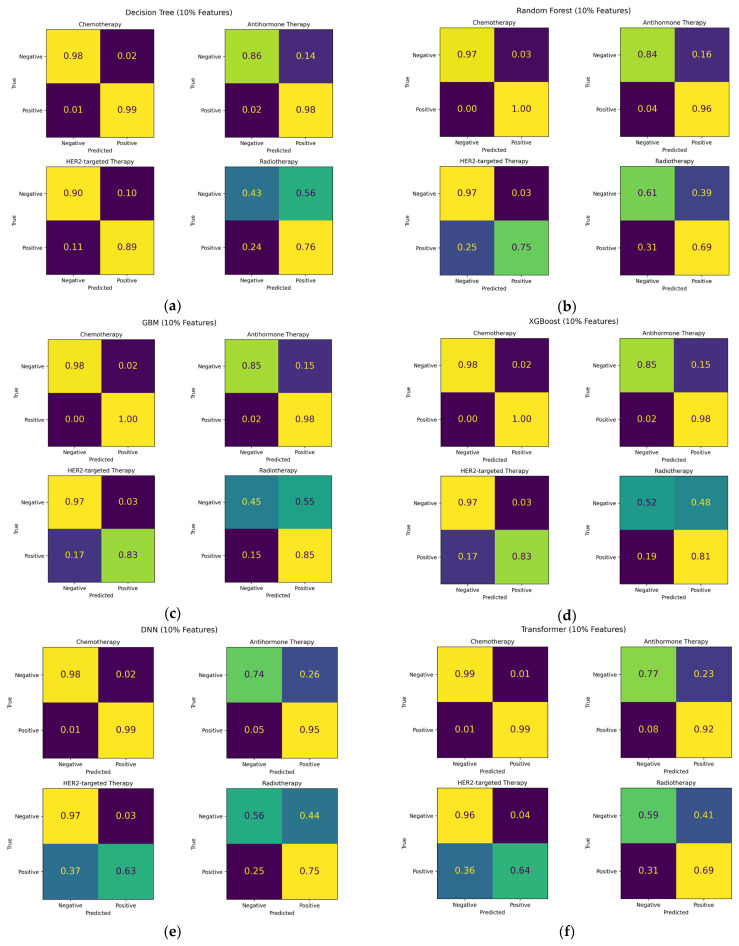
Confusion matrices for breast cancer treatment classification using the top 10% feature subset: (**a**) Decision Tree, (**b**) Random Forest, (**c**) GBM, (**d**) XGBoost, (**e**) DNN, and (**f**) Transformer. The confusion matrices are presented in terms of true positive rate (TPR), false positive rate (FPR), true negative rate (TNR), and false negative rate (FNR).

**Figure 9:**
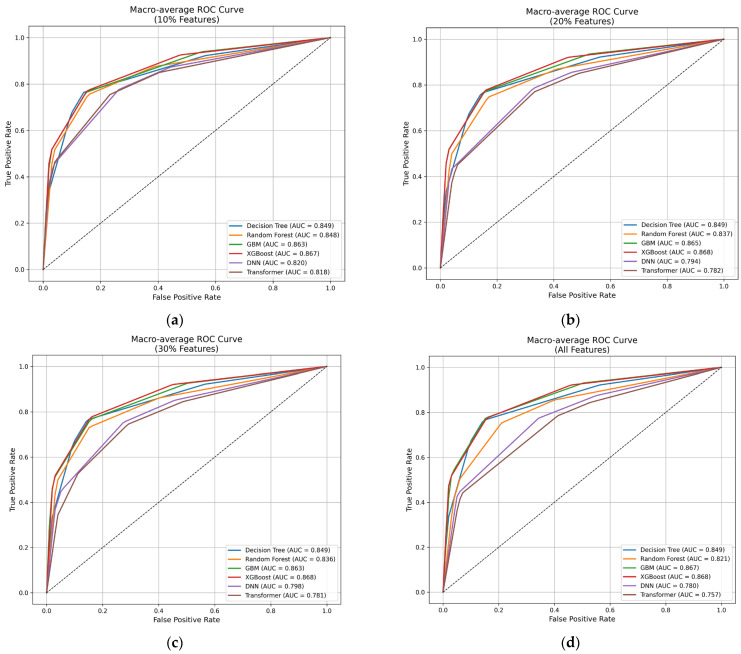
Receiver operating characteristic (ROC) curves of the six classification models trained on (**a**) top 10% of selected features, (**b**) top 20% of selected features, (**c**) top 30% of selected features, and (**d**) full feature set for multi-label breast cancer treatment prediction. The area under the curve (AUC) values demonstrate that ensemble-based models, particularly Gradient Boosting Machine (GBM) and XGBoost, achieved a higher discrimination performance compared with the other models.

A comparative evaluation was conducted to assess the performance of the six models—Decision Tree, Random Forest, GBM, XGBoost, DNN, and Transformer—used for the multi-label classification of breast cancer treatment strategies. Each model was trained and evaluated using the full feature set and three feature subsets corresponding to the top 10%, 20%, and 30% of feature importance scores. Figure 8 shows the confusion matrices of the six classification models evaluated using the top 10% feature subset. Among the models, Gradient Boosting Machine (GBM) and XGBoost indicate more accurate class-wise predictions compared with Random Forest and other models. In particular, XGBoost showed the most consistent separation of major treatment classes in the test set, whereas Random Forest demonstrated increased confusion among minority classes. Based on these confusion matrix results, the receiver operating characteristic (ROC) curves of the six models were trained on feature subsets as well as on all features, as shown in Figure 9. Across all feature subsets, the GBM and XGBoost models achieved the highest area under the curve (AUC) values. The Decision Tree and Random Forest models showed moderate performance, with AUC values around 0.84 to 0.85. By contrast, the DNN and Transformer models recorded lower AUC values, which were generally below 0.82.

The results were assessed using both macro-averaged and micro-averaged performance metrics to provide a comprehensive understanding of model behavior from different feature sets. Table 8 demonstrates the macro-averaged recall, precision, and F1 scores obtained from six classification models evaluated on feature subsets and the full feature set. The ensemble-based models, GBM and XGBoost, achieved the highest performance across all feature subsets, with macro-averaged F1 scores around 0.88. The Decision Tree and Random Forest models showed a stable but slightly lower performance, maintaining F1 scores of approximately 0.84–0.86. By contrast, the DNN and Transformer models exhibited relatively lower accuracy, especially when the full feature set was used. This result indicates that neural network-based approaches were less suitable for structured tabular data without strong contextual or sequential dependencies.

Table 9 shows the micro-averaged results for the same models and feature sets. Overall, the ranking of model performance remained consistent with the macro-averaged results. GBM and XGBoost again demonstrated the highest predictive performance, achieving micro-averaged F1 scores close to 0.9. The micro-averaged values were slightly higher than the macro-averaged because they were influenced by frequent treatment combinations that appeared more often in the dataset.

When comparing the two evaluation methods, macro-averaging provided a more balanced and reliable assessment for this dataset. Since the dataset contained imbalanced distributions across the 16 treatment combinations, the macro-averaging approach was selected as the primary evaluation metric. This method ensured that rare but clinically important treatment classes contributed equally to model evaluation. The strong and consistent macro-averaged results demonstrated the robustness of the proposed framework under class-imbalanced conditions, which reflects its potential applicability in real-world clinical environments where such imbalance is common.

Overall, the ensemble-based models demonstrated stable predictive accuracy across different feature subset sizes. These findings indicate that GBM and XGBoost provide the most effective and balanced predictive performance for multi-label classification in breast cancer treatment decision-making, supporting their suitability for a CDSS using structured pathological and clinical data.

## 4. Discussion

Breast cancer is a highly prevalent and biologically heterogeneous disease that requires accurate staging to guide diagnosis and treatment. Staging depends on key pathological factors, such as tumor size, lymph node involvement, and distant metastasis, which are essential for determining the prognosis and for planning the individualized therapies. Although pathology reports play a critical role in determining appropriate treatment strategies for breast cancer, their interpretation is often difficult because clinicians must integrate complex pathological findings with other medical information. This process becomes increasingly time-consuming when managing a large number of patients, and is often prone to transcription or judgment errors, which can ultimately affect the consistency and reliability of the treatment decisions. Furthermore, variations in reporting formats across institutions may contribute to increased complexity and cost.

This study aimed to develop an integrated CDSS for breast cancer treatment by combining LLM-based pathology report analysis with deep learning-based predictive modeling. The comparative evaluation of six classification models demonstrated that ensemble-based approaches, particularly GBM and XGBoost, achieved the most consistent and superior predictive performance across both macro- and micro-averaged evaluations. These models exhibited strong robustness and interpretability when applied to structured clinical and pathological datasets, suggesting their suitability for real-world CDSS applications. However, deep learning-based models, such as DNN and Transformer, showed relatively lower accuracy, primarily because they are optimized for sequential or contextual data, whereas the dataset used in this study consisted mainly of structured tabular variables. In addition to data structure, label imbalance likely contributed to the relatively lower performance of the DNN and Transformer models. The 16 treatment combination classes in this study exhibited substantial imbalance, with several rare treatment patterns represented by a limited number of samples. Such imbalance can hinder deep learning models, which typically require large and well-balanced datasets to effectively learn complex label dependencies in a multi-label classification. Although Transformer architectures excel at modeling sequential or contextual information, they do not inherently address class imbalance in structured tabular data. By contrast, ensemble-based models, such as Gradient Boosting Machine (GBM) and XGBoost, are more robust to imbalanced class distributions due to their tree-based learning and iterative optimization mechanisms, which explains their more stable and superior performance in this study. This finding indicates that model selection must be guided by the fundamental characteristics of the medical data, including label imbalance and structured feature representations, rather than relying solely on the complexity or novelty of neural network architecture.

Recent studies have shown that Transformer-based models using clinical risk factors are effective for breast cancer risk prediction [37], and ensemble self-attention Transformer frameworks applied to mammography images have demonstrated strong performance in breast cancer diagnosis [38]. However, our study extends these approaches by integrating LLM-based pathology report extraction with structured clinical data to support treatment decision-making rather than risk prediction or image-based diagnosis.

Furthermore, the experimental results showed that the classification performance exhibited no substantial difference among the top 10%, 20%, and 30% feature subsets and the full feature set. This finding indicates that expanding the feature space did not yield any noticeable improvement in the predictive performance. Using a smaller number of features also reduces computational cost and analysis time; therefore, selecting key clinical factors that significantly influence breast cancer treatment decisions can be considered to be an effective approach to achieving reliable predictive performance with enhanced time and cost efficiency.

Collectively, these findings provide a solid foundation for the development of an AI-driven CDSS that can effectively integrate structured clinical and pathological information to support reliable and data-informed treatment decision-making in breast cancer care. Despite these promising results, this study has several limitations. First, patients with stage IV breast cancer were excluded from the analysis because treatment decision-making for metastatic disease is influenced by diverse and complex factors beyond the molecular subtype, including the extent of metastasis, previous therapy, and patient condition. Second, the current study focused solely on the pathological data obtained after surgery, without considering preoperative clinical conditions. In contemporary clinical practice, neoadjuvant chemotherapy is increasingly performed prior to surgery, which often alters the pathological findings relative to the pre-treatment state. Therefore, future studies should integrate multimodal data—including imaging and preoperative clinical information—to improve the clinical relevance of the model. Third, adjuvant therapy decisions may vary according to histological subtypes with favorable prognostic features, regardless of the TNM stage or immunohistochemical status. Moreover, treatment guidelines continue to evolve, requiring continuous model refinement and validation.

Nevertheless, the AI-based CDSS developed in this study demonstrates significant clinical utility. By automating the extraction and integration of complex pathological and clinical information, the system can reduce clinician workload, minimize human error, and improve efficiency in treatment planning. Furthermore, it can support less experienced clinicians in making accurate and standardized treatment decisions aligned with well-established standardized guidelines, thereby enhancing consistency and reliability in clinical practice. Furthermore, by integrating structured clinical data with the staging information extracted from the pathology reports, the system promotes evidence-based and data-driven decision-making, supporting the broader application of precision medicine in real-world oncology settings.

Future research will integrate multicenter datasets to enhance the generalizability and clinical applicability of the proposed system and to validate its performance using diverse real-world data. In addition, larger and more representative cohorts will be incorporated to better reflect the complexity of treatment options encountered in routine clinical practice. Finally, a web-based, interactive AI-driven decision support system will be developed to provide clinicians with real-time and explainable treatment recommendations.

Breast cancer treatment has become increasingly complex due to rapid advances. The pace of these developments has accelerated to the extent that major clinical guidelines are revised frequently, which makes it difficult for clinicians to keep up with the latest evidence through traditional textbook-based learning alone. As treatment options continue to diversify, direct comparisons among the individual therapies become more challenging, which complicates the selection of optimal treatment strategies in routine clinical practice. This complexity may result in substantial variability in treatment decisions among specialists, trainees, and community practitioners, and it may impose considerable cognitive burden and burnout even for experienced clinicians. In this context, our study explores the role of artificial intelligence as an assistive decision-support tool that helps synthesize pathological and clinical information, rather than serving as a replacement for clinical judgment. Given the potential risks associated with uncritical AI use, careful validation and clinician oversight are essential to ensure responsible clinical application.

## 5. Conclusions

This study developed an integrated CDSS for breast cancer treatment by combining the LLM-based extraction of pathological staging information with machine learning and the deep learning-based prediction of treatment strategies. Among the six evaluated models, ensemble-based algorithms, such as GBM and XGBoost, achieved the highest and most consistent performance in predicting treatment combinations from structured pathological and clinical data. The findings show that integrating LLM-derived pathology interpretation with data-driven predictive modeling improves the accuracy and reliability of treatment decision support in oncology. This research demonstrates that artificial intelligence can play a critical role in supporting evidence-based and consistent clinical decision-making for breast cancer management.

## Figures and Tables

**Table 1:** Distribution of tables in the database for the study population. Data were extracted across 12 categories directly from the electronic medical records of the hospital.

Category	Number of Records	Number of Patients
Patient Information	6175	6175
Outpatient Visits	574,364	6171
Inpatient Visits	27,350	6008
Diagnosis	877,357	6175
Drug Prescriptions	2,092,681	6143
Surgical Prescriptions	23,011	5685
Treatments and Procedures	748,103	6169
Lab Tests	405,279	6156
Imaging and Pathology	354,955	6152
Diagnostic Tests	6,524,055	6154
Physical Measurements	55,572	5980
Vital Signs	1,947,671	5932

**Table 2:** Multi-class label configuration for breast cancer treatment prediction.

Class No.	Chemo- Therapy	Anti- Hormone Therapy	HER2- Targeted Therapy	Radio Therapy	Description
0	✗	✗	✗	✗	None
1	✓	✗	✗	✗	Chemotherapy only
2	✗	✓	✗	✗	Anti-hormone therapy only
3	✗	✗	✓	✗	HER2-targeted therapy only
4	✗	✗	✗	✓	Radiotherapy only
5	✓	✓	✗	✗	Chemotherapy + Anti-hormone therapy
6	✓	✗	✓	✗	Chemotherapy + HER2-targeted therapy
7	✓	✗	✗	✓	Chemotherapy + Radiotherapy
8	✗	✓	✓	✗	Anti-hormone + HER2-targeted therapy
9	✗	✓	✗	✓	Anti-hormone + Radiotherapy
10	✗	✗	✓	✓	HER2-targeted + Radiotherapy
11	✓	✓	✓	✗	Chemotherapy + Anti-hormone + HER2-targeted therapy
12	✓	✓	✗	✓	Chemotherapy + Anti-hormone + Radiotherapy
13	✓	✗	✓	✓	Chemotherapy + HER2-targeted + Radiotherapy
14	✗	✓	✓	✓	Anti-hormone + HER2-targeted + Radiotherapy
15	✓	✓	✓	✓	Chemotherapy + Anti-hormone + HER2-targeted + Radiotherapy

**Table 3:** Hyperparameters of the Random Forest model for feature selection.

Model	Parameters	Hyperparameter Values		
Random Forest	n_estimators	300	500	700
	max_depth	5	10	15
	min_samples_split	2	5	10
	min_samples_leaf	1	2	4

**Table 4:** Hyperparameter configuration for each model.

Model	Parameters	Hyperparameter Values		
Decision Tree	criterion	gini	entropy	-
	max_depth	5	10	15
	min_samples_split	2	5	10
	min_samples_leaf	1	2	4
Random Forest	n_estimators	300	500	700
	max_depth	5	10	15
	min_samples_split	2	5	10
	min_samples_leaf	1	2	4
GBM	n_estimators	300	500	700
	max_depth	5	10	15
	learning_rate	0.1	0.05	0.01
	subsample	0.8	1.0	-
XGBoost	n_estimators	300	500	700
	max_depth	5	10	15
	learning_rate	0.1	0.05	0.01
	min_child_weight	3	5	7
DNN	epochs	500	1000	3000
	hidden_dim	64	128	256
	learning_rate	0.001	0.0005	0.0001
	dropout	0.1	0.2	0.4
Transformer	epochs	500	1000	3000
	d_model	64	128	-
	learning_rate	0.001	0.0005	0.0001
	nhead	2	4	8

**Table 5:** Distribution of breast cancer patients by age and clinical stage.

Age	Stage 0	Stage IA	Stage IB	Stage IIA	Stage IIB	Stage IIIA	Stage IIIB	Stage IIIC
[10, 20)	1	0	0	1	0	0	0	0
[20, 30)	4	12	0	7	4	1	0	0
[30, 40)	28	126	1	115	70	42	3	13
[40, 50)	98	659	6	412	239	135	8	40
[50, 60)	87	642	8	400	202	114	6	49
[60, 70)	48	438	8	273	128	67	4	35
[70, 80)	19	146	0	132	55	36	3	17
[80, 90)	2	28	0	17	12	9	3	0
[90, 100)	0	0	0	1	0	0	0	1
Total	287	2051	23	1358	710	404	27	155

**Table 6:** Distribution of patients across 16 treatment combination classes.

Class No.	Chemotherapy	Anti-Hormone Therapy	HER2-Targeted Therapy	Radiotherapy	Number of Patients
0	0	0	0	0	198
1	1	0	0	0	306
2	0	1	0	0	623
3	0	0	1	0	1
4	0	0	0	1	86
5	1	1	0	0	579
6	1	0	1	0	159
7	1	0	0	1	318
8	0	1	1	0	3
9	0	1	0	1	1001
10	0	0	1	1	0
11	1	1	1	0	191
12	1	1	0	1	1078
13	1	0	1	1	191
14	0	1	1	1	2
15	1	1	1	1	279

**Table 7:** Sample distribution of classes after dataset splitting.

Class	Training Set	Validation Set	Test Set	Total Number of Samples
0	116	38	44	198
1	196	49	61	306
2	368	137	118	623
3	1	0	0	1
4	51	18	17	86
5	361	109	109	579
6	107	25	27	159
7	189	59	70	318
8	2	0	1	3
9	600	201	200	1001
10	0	0	0	0
11	96	52	43	191
12	639	226	213	1078
13	113	38	40	191
14	2	0	0	2
15	168	51	60	279

**Table 8:** Macro-averaged recall, precision, and F1 score across feature selection subsets (top 10%, 20%, 30%) and the full feature set.

Rate of Features	Decision Tree			Random Forest			GBM			XGBoost			DNN			Transformer		
	Recall	Precision	F1 Score	Recall	Precision	F1 Score	Recall	Precision	F1 Score	Recall	Precision	F1 Score	Recall	Precision	F1 Score	Recall	Precision	F1 Score
10%	0.85 ± 0.03	0.83 ± 0.01	0.84 ± 0.01	0.83 ± 0.03	0.86 ± 0.03	0.84 ± 0.01	0.89 ± 0.02	0.87 ± 0.02	0.88 ± 0.02	0.89 ± 0.0	0.87 ± 0.0	0.88 ± 0.0	0.8 ± 0.01	0.85 ± 0.01	0.82 ± 0.01	0.81 ± 0.01	0.82 ± 0.01	0.81 ± 0.01
20%	0.86 ± 0.03	0.83 ± 0.01	0.84 ± 0.01	0.82 ± 0.03	0.85 ± 0.03	0.83 ± 0.02	0.89 ± 0.02	0.87 ± 0.02	0.88 ± 0.02	0.89 ± 0.01	0.87 ± 0.0	0.88 ± 0.0	0.78 ± 0.01	0.82 ± 0.01	0.8 ± 0.01	0.78 ± 0.01	0.79 ± 0.01	0.79 ± 0.01
30%	0.86 ± 0.03	0.83 ± 0.01	0.84 ± 0.01	0.81 ± 0.03	0.85 ± 0.03	0.82 ± 0.02	0.89 ± 0.02	0.87 ± 0.02	0.88 ± 0.01	0.89 ± 0.01	0.87 ± 0.0	0.88 ± 0.0	0.77 ± 0.01	0.8 ± 0.01	0.78 ± 0.01	0.76 ± 0.01	0.77 ± 0.01	0.76 ± 0.01
All	0.86 ± 0.03	0.83 ± 0.01	0.84 ± 0.01	0.8 ± 0.03	0.82 ± 0.04	0.8 ± 0.02	0.89 ± 0.02	0.87 ± 0.01	0.88 ± 0.01	0.89 ± 0.01	0.87 ± 0.0	0.88 ± 0.0	0.75 ± 0.03	0.79 ± 0.02	0.76 ± 0.02	0.74 ± 0.02	0.75 ± 0.01	0.74 ± 0.01

**Table 9:** Micro-averaged recall, precision, and F1 score across feature selection subsets (top 10%, 20%, 30%) and the full feature set.

Rate of Features	Decision Tree			Random Forest			GBM			XGBoost			DNN			Transformer		
	Recall	Precision	F1 Score	Recall	Precision	F1 Score	Recall	Precision	F1 Score	Recall	Precision	F1 Score	Recall	Precision	F1 Score	Recall	Precision	F1 Score
10%	0.87 ± 0.02	0.86 ± 0.01	0.87 ± 0.01	0.87 ± 0.01	0.88 ± 0.01	0.88 ± 0.01	0.92 ± 0.01	0.88 ± 0.01	0.9 ± 0.01	0.91 ± 0.01	0.88 ± 0.0	0.9 ± 0.0	0.87 ± 0.01	0.87 ± 0.01	0.87 ± 0.0	0.85 ± 0.01	0.86 ± 0.01	0.86 ± 0.01
20%	0.88 ± 0.02	0.86 ± 0.01	0.87 ± 0.01	0.87 ± 0.01	0.87 ± 0.01	0.87 ± 0.01	0.91 ± 0.01	0.88 ± 0.01	0.89 ± 0.01	0.91 ± 0.01	0.88 ± 0.0	0.9 ± 0.0	0.85 ± 0.01	0.85 ± 0.01	0.85 ± 0.0	0.84 ± 0.01	0.83 ± 0.0	0.84 ± 0.01
30%	0.88 ± 0.02	0.86 ± 0.01	0.87 ± 0.01	0.87 ± 0.01	0.87 ± 0.01	0.87 ± 0.01	0.91 ± 0.01	0.88 ± 0.01	0.9 ± 0.01	0.91 ± 0.01	0.88 ± 0.0	0.9 ± 0.0	0.84 ± 0.02	0.84 ± 0.01	0.84 ± 0.01	0.82 ± 0.01	0.82 ± 0.0	0.82 ± 0.01
All	0.88 ± 0.02	0.86 ± 0.01	0.87 ± 0.01	0.86 ± 0.01	0.86 ± 0.02	0.86 ± 0.01	0.92 ± 0.01	0.88 ± 0.01	0.9 ± 0.01	0.91 ± 0.01	0.88 ± 0.0	0.9 ± 0.0	0.82 ± 0.03	0.82 ± 0.01	0.82 ± 0.02	0.78 ± 0.03	0.8 ± 0.01	0.79 ± 0.02

## Data Availability

The datasets generated and/or analyzed during the current study are not publicly available due to the hospital policy but are available from the corresponding author upon reasonable request.
